# Bile Salt-Stimulated Lipase Plays an Unexpected Role in Arthritis Development in Rodents

**DOI:** 10.1371/journal.pone.0047006

**Published:** 2012-10-11

**Authors:** Susanne Lindquist, Eva-Lotta Andersson, Lennart Lundberg, Olle Hernell

**Affiliations:** Department of Clinical Sciences, Pediatrics, Umeå University, Umeå, Sweden; University Hospital Jena, Germany

## Abstract

**Objective:**

The present study aimed to explore the hypothesis that bile salt-stimulated lipase (BSSL), in addition to being a key enzyme in dietary fat digestion during early infancy, plays an important role in inflammation, notably arthritis.

**Methods:**

Collagen-induced arthritis (CIA) and pristane-induced arthritis (PIA) in rodents are commonly used experimental models that reproduce many of the pathogenic mechanisms of human rheumatoid arthritis, i.e. increased cellular infiltration, synovial hyperplasia, pannus formation, and erosion of cartilage and bone in the distal joints. We used the CIA model to compare the response in BSSL wild type (BSSL-WT) mice with BSSL-deficient ‘knock-out’ (BSSL-KO) and BSSL-heterozygous (BSSL-HET) littermates. We also investigated if intraperitoneal injection of BSSL-neutralizing antibodies affected the development or severity of CIA and PIA in mice and rats, respectively.

**Results:**

In two consecutive studies, we found that BSSL-KO male mice, in contrast to BSSL-WT littermates, were significantly protected from developing arthritis. We also found that BSSL-HET mice were less prone to develop disease compared to BSSL-WT mice, but not as resistant as BSSL-KO mice, suggesting a gene-dose effect. Moreover, we found that BSSL-neutralizing antibody injection reduced both the incidence and severity of CIA and PIA in rodents.

**Conclusion:**

Our data strongly support BSSL as a key player in the inflammatory process, at least in rodents. It also suggests the possibility that BSSL-neutralizing agents could serve as a therapeutic model to reduce the inflammatory response in humans.

## Introduction

Collagen-induced arthritis (CIA) in mice is a commonly used experimental model that reproduces many of the pathogenic features of human rheumatoid arthritis (RA), i.e. increased infiltration of neutrophilic granulocytes, synovial hyperplasia, pannus formation, and erosion of cartilage and bone in the distal joints. In the present study we used the CIA model to test the hypothesis that bile salt-stimulated lipase (BSSL), also termed carboxyl ester lipase or bile salt-dependent lipase, is a key component of inflammation, including chronic arthritis. BSSL is primarily recognized as a lipolytic enzyme that facilitates digestion and absorption of dietary fat. It has broad specificity and hydrolyzes a variety of different substrates [1]–[3]. BSSL is expressed in the exocrine pancreas and secreted into the intestinal lumen in all species thus far investigated, including those devoid of pancreatic triglyceride lipase [4], [5]. Besides BSSL an oncofetal variant termed feto-acinar pancreatic protein (FAPP), has been described. This form is poorly secreted and exclusively expressed in human fetal and diseased pancreas, but to our knowledge not in other species, or in pancreas of healthy adults [6].

In some species, including humans, BSSL is also expressed by the lactating mammary gland and secreted in milk. Milk-derived BSSL contributes significantly to the efficient utilization of milk fat in breastfed infants [7], [8]. In fact, together with pancreatic lipase related protein 2, BSSL is the key enzyme in neonatal intestinal fat digestion [9]–[11].

BSSL may also have effects beyond the gastrointestinal tract. It is present in the blood [12], but so far the source of circulating BSSL is uncertain. While it has been suggested that circulating BSSL originates in the pancreas and reaches the intravascular space via intestinal absorption and passage through the arterial wall [13], some authors suggest that BSSL is expressed and secreted by macrophages [14] and endothelial cells [15]. Contrasting to the view that BSSL is absorbed by the intestine, we and others have shown that neither does serum levels of BSSL increase after a meal of breast milk, nor do serum levels of BSSL in breastfed and formula-fed human infants differ, even though breast milk is the major source of BSSL in the breastfed newborn but is absent from formula [16], [17]. With respect to function, some argue that circulating BSSL influences lipoprotein metabolism, chylomicron assembly and secretion, reverse cholesterol transport, and modulates atherosclerosis [2], [18]–[20]. We showed that BSSL prevents binding of HIV-1 to its receptor on dendritic cells *in vitro*, and thereby inhibits HIV-1 transfer to CD4^+^ T-cells [21], [22]. BSSL was also found to be stored in platelets and released upon their activation, suggesting a role in thrombus formation [23]. In the latter study, released BSSL was proposed to interact with the chemokine receptor CXCR4 on platelets to enhance platelet aggregation and thrombus formation. Recently, we discovered that BSSL is expressed by polymorphonuclear granulocytes present in human liver biopsies and in circulating granulocytes collected from healthy volunteers (Lindquist et al., unpublished). Collectively these observations suggested that BSSL could be a common denominator in inflammation and thus play an important role in the inflammatory process.

We here report that BSSL deficient mice are significantly less prone to develop disease in the CIA model compared to wild type littermates. Moreover, we show that administration of BSSL neutralizing antibodies suppresses the course of disease in CIA mice and also in pristane induced arthritis (PIA) in rats, another commonly used RA model [24].

## Materials and Methods

### Experimental animals

Female DBA/1 mice, aged 10–12 weeks at arrival, were purchased from Taconic Europe (Ejby, Denmark). DBA/1 mice are widely used for CIA studies and although male DBA/1 mice may be slightly more susceptible to develop arthritis compared to female DBA/1 mice, we used females for therapeutic trials. The advantage being that females can be randomly mixed in cages during experiments, while males tend to fight if they are put together.

Male Dark Agouti (DA) rats, aged 8–10 weeks at arrival, were purchased from Harlan Laboratories Europe (Horst, The Netherlands). BSSL knock-out (BSSL-KO) mice of C57BL/6J background have been described previously [25] and were a kind gift from Dr. J. Breslow, Rockefeller University, New York. To obtain susceptibility to CIA, conferred by the major histocompatibility complex (MHC) A^q^ haplotype [26], BSSL-KO mice were crossed to mouse strain C57BL/10Q (obtained from the Medical Inflammation Research, Lund University, Lund, Sweden) initially for one generation. BSSL heterozygous (BSSL-HET) F1 offspring were then inter-crossed to generate BSSL-KO and BSSL wild type (BSSL-WT) littermates that were further screened and selected as either homozygous or heterozygous for the MHC A^q^ allele. The mice included in the first CIA experiment were 13 BSSL-KO/A^q^ homozygous (9 males, four females), 23 BSSL-KO/A^q^ heterozygous (8 males, 15 females), 12 BSSL-WT/A^q^ homozygous (8 males, four females) and 16 BSSL-WT/A^q^ heterozygous (8 males, 8 females). All experiments were performed on littermates to exclude nonlinked genetic effects. After the initial CIA experiment, the BSSL-KO allele was backcrossed to mouse strain C57BL/10Q for seven generations. BSSL-HET/MHC A^q^ homozygous mice were then inter-crossed to generate BSSL-KO, BSSL-HET and BSSL-WT littermates that were used in following CIA experiment. Screening of BSSL genotypes was performed by polymerase chain reaction (PCR) as previously described [25] and MHC genotyping was performed using microsatellite marker D17mit230 [27].

All mice and rats were bred or kept at 12 h light/dark cycles in polystyrene cages containing wood shavings and were fed with standard rodent chow and water ad libitum at the common animal facilities of Umeå University, Umeå (mice) or Biomedical Centre, Lund University, Lund (rats).

### Ethics Statement

Mice experiments were approved by the Umeå ethical committee (permission Nos. A61-08 and A103-10) and rat experiments were approved by the Malmö/Lund ethical committee (permission No. M107-07).

### Collagen-induced arthritis

CIA was induced in mice according to standard protocol [28]. In short, mice were immunized by subcutaneous (s.c.) injection at the base of the tail with 100 µg rat collagen type II (CII), prepared as described [29], or chick CII (Chondrex, Inc. Redmond, WA, USA), each emulsified in an equal volume of complete Freund's adjuvant (CFA; Sigma-Aldrich, St. Louis, MO, USA) at a final concentration of 2 mg/ml. After 21 days, mice were boosted by injection near the first injection site with 50 µg rat or chick CII emulsified in equal volume of incomplete Freund's adjuvant (IFA; Sigma-Aldrich) at final concentration of 1 mg/ml. Severity of disease was followed by clinical scoring every second or third day, starting one week before boost.

### Clinical evaluation of arthritis

Development of arthritis was evaluated using a macroscopic scoring system based on the number of inflamed joints (defined by swelling and redness of the joint) in each paw. Each inflamed digit and knuckle scores 1 point each, whereas an inflamed wrist or ankle scores 5 points, resulting in a maximum score of 15 for each paw and 60 for each mouse [30]. To avoid cage-depending variation, mice or rats of different genotypes or from different treatment groups were always randomly mixed in cages during the experiments. The examiner was blinded to BSSL genotypes or treatment group during scoring.

### Pristane-induced arthritis

PIA was induced in rats as previously described [24], [31]. In brief, DA rats, known to have a high susceptibility for developing PIA, were given a single s.c. injection of 150 µl pristane (Acros Organics, Geel, Belgium) at the base of the tail. Development of disease (arthritis severity) was followed by clinical scoring every second to third day as described above.

### Antibodies

Monospecific rabbit anti-mouse BSSL antibodies (CDV15) were developed by Agrisera AB (Vännäs, Sweden). The CDV15 antibodies were raised in rabbits by immunization with a synthetic peptide corresponding to amino acid 328–341 in the mature mouse protein [32]. Polyclonal anti-peptide 328–341 antibodies were purified from serum using a peptide affinity column obtained from Agrisera AB according to the suppliers' protocol. Normal rabbit IgG (purified on protein G sepharose) was obtained from Agrisera AB. CDV15 antibodies and normal rabbit IgG were diluted in phosphate-buffered saline (PBS; Gibco-Invitrogen, Carlsbad, CA, USA) and passed through a 0.45 µm sterile filter before use.

### Western blot analysis

Pieces of pancreatic tissue obtained from mice and rats were homogenized in APBS buffer (137 mM NaCl, 2.7 mM KCl, 4.3 mM Na_2_HPO_4_, 1.4 mM KH_2_PO_4_, pH 7.0) containing 1 Mini Complete tablet per 10 ml (Roche Diagnostics, Mannheim, Germany). The homogenate was centrifuged at 14,000 rpm for 10 min and the supernatant was collected. All steps were performed at 4°C to minimize the risk of proteolysis. Approximately 30 µg of total protein was separated on 12% SDS- polyacrylamide gel electrophoresis according to Laemmli [33] and transferred to polyvinylidene difluoride (PVDF) membranes (Bio-Rad, Hercules, CA). Western blotting was carried out using the ECL Advance Western Blotting Detection Kit, following the manufacturer's recommendations (GE Healthcare). The CDV15 antibody was used as primary antibody, and a peroxidase-conjugated donkey-anti-rabbit IgG (DAKO, Glostrup, Denmark) was used as secondary antibody.

### Treatment of collagen-induced arthritic mice with BSSL neutralizing antibodies

Seventy-five female DBA/1 mice (Taconic, 12 weeks of age) were randomized to five groups of 15 mice each for treatment. CIA was induced in all mice by an injection of chick CII at day 0 and a booster dose given on day 21. Every fourth day, beginning the day before boost (day 20), each group was given an intraperitoneal (i.p.) injection (0.2 ml total volume) with one of the following: 0.1 mg CDV15 antibody, 0.2 mg CDV15 antibody, 0.1 mg normal rabbit IgG, 0.2 mg normal rabbit IgG, or PBS (Gibco). Development of disease (arthritis severity) was followed by clinical scoring every fourth day, starting at day 20.

### Treatment of pristane-induced arthritic rats with BSSL neutralizing antibodies

Forty DA rats were injected with pristane to induce arthritis at day 0. The rats were randomized to one of four groups of 10 animals each for treatment. At day 5, 10, and 15 the rats were treated by i.p. injection (1 ml) with one of the following; anti-BSSL 1 mg/kg, anti-BSSL 5 mg/kg, etanercept (Enbrel, Wyeth, Great Britain) 1 mg/kg, or PBS (Gibco). Development of disease (arthritis severity) was followed by clinical scoring daily or every second day as described above, starting on day 8.

### Plasma anti-CII antibody concentration

The antibody response against rat CII in plasma was determined using enzyme-linked immunosorbent assay (ELISA) as described [34]. Briefly, 96-well plates (Costar, Cambridge, MA, USA) were coated overnight at 4°C with 50 µl per well of 10 µg/ml rat CII in PBS. All washes were performed with PBS (pH 7.4) containing 0.1% Tween-20. Plasma was diluted in PBS and analyzed in duplicates. The amount of bound IgG antibody was estimated after incubation with biotin-conjugated isotype-specific antibodies (Southern Biotechnology Associates, Inc. Birmingham, AL, USA) followed by Extravidin-Peroxidase (Sigma) and developed with ABTS (Roche Diagnostics GmbH, Mannheim, Germany) as substrate followed by detection in a Spectra Max at OD 405 nm (Molecular Devices, Sunnyvale, CA, USA).

### Plasma cartilage oligomeric matrix protein concentration

Plasma concentration of cartilage oligomeric matrix protein (COMP) was determined by competitive ELISA as described [35]. Briefly, rat COMP was used for coating of the microtiter plates and for preparing the standard curve included on each plate. Plates were blocked with 1% bovine serum albumin (BSA) in PBS for 2 hours at room temperature. After blocking, plasma co-incubated with rabbit polyclonal antiserum against rat COMP was added and the plates were incubated for 2 hours at room temperature. The amount of plasma COMP was estimated after incubation with an alkaline phosphatase-conjugated swine-anti-rabbit isotype-specific antibody (DAKO, Glostrup, Denmark) and phosphatase substrate (Sigma Aldrich) followed by detection in a Spectra Max (Molecular Devices) at OD 405 nm.

### Histological examination

Hind limbs were removed from representative animals after the animals were killed at study termination. The samples were fixed in 4% phosphate buffered paraformaldehyde for 24 hours, decalcified in 1% EDTA solution, and embedded in paraffin. All samples were sectioned and stained with hematoxylin and eosin at Histo-Center AB (Göteborg, Sweden).

### Statistical analyses

Statistical analyses were performed using SPSS 20.0 (SPSS Inc., Chicago, IL, USA). The Mann-Whitney U test (exact routine) was used to compare mean arthritis scores and serum concentrations of COMP, anti-CII antibody, and anti-rabbit IgG between BSSL-KO versus BSSL-WT mice. To compare mean arthritis scores and anti-rabbit IgG concentrations between multiple groups of mice, the Kruskal-Wallis exact test was used; in case of an overall significant difference, pair wise Mann-Whitney U tests were subsequently performed. Derived p-values were then adjusted for multiple testing (Bonferroni correction). Fisher's exact test was used to compare the cumulative incidence of arthritis between BSSL-KO and BSSL-WT mice, or between multiple groups of mice. In the latter case, derived p-values were adjusted for multiple testing. Adjusted p-values of <0.05 were considered statistically significant.

## Results

### BSSL deficient mice are resistant to collagen induced arthritis

**Figure 1:**
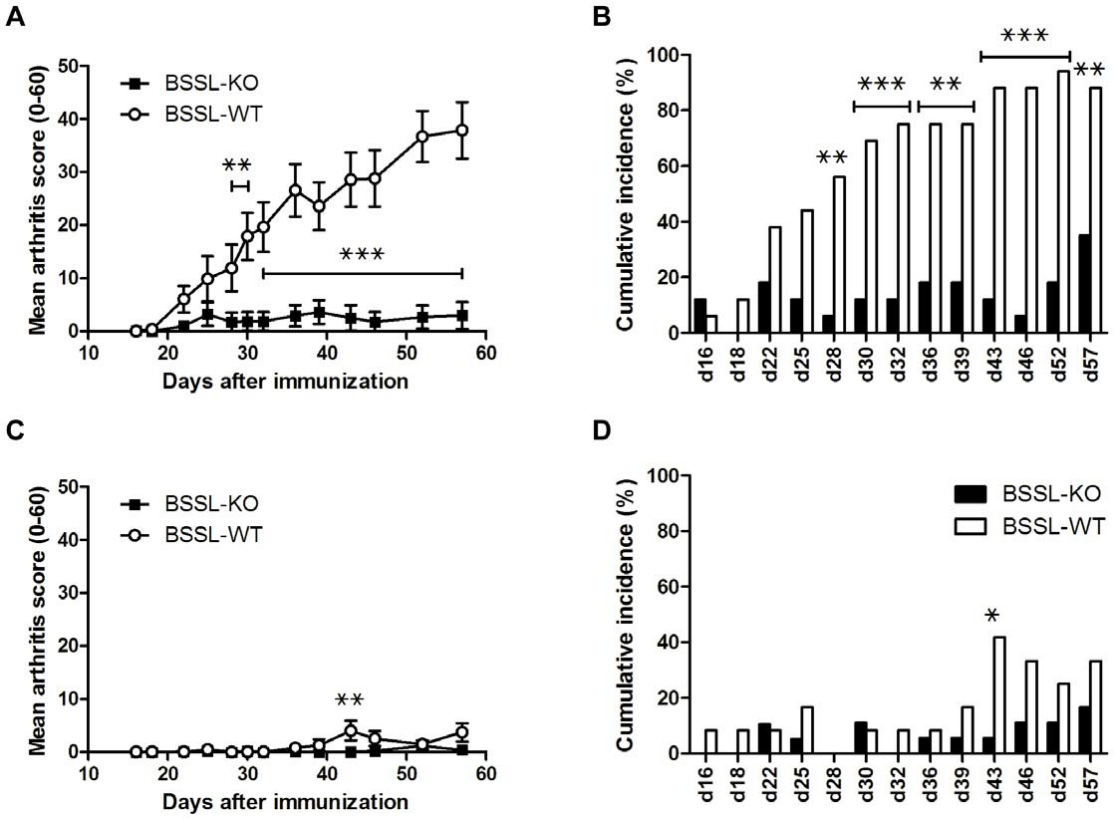
Collagen-induced arthritis in BSSL-KO and BSSL-WT mice. Littermates of BSSL-KO and BSSL-WT mice were immunized with rat collagen type II (CII) on day 0 and boosted on day 21. Arthritis development was followed by blinded clinical scoring 2–3 times per week for 57 days after the initial CII injection. Mean clinical score of arthritis severity in (A) male mice and (C) female mice and the cumulative incidence of arthritis presented as percentage of affected mice with clinical score≥1 in (B) male mice and (D) female mice are shown. Error bars indicate ± SEM. ??^???^ = p<0.05; ^???^ = p<0.01; ^???^ = p<0.001.

The susceptibility of BSSL-KO mice (17 males and 19 females, age 8–11 weeks) to develop CIA was compared to that of BSSL-WT littermates (16 males and 12 females) and immediately a distinct gender-based difference was found. The most striking result was detected in BSSL deficient male mice, which were almost completely protected from arthritis, and consequently developed significantly lower mean arthritis scores compared to their BSSL-WT male littermates (p<0.01 from day 28 onwards; Fig. 1A). The proportion (cumulative incidence) of animals that developed disease (arthritis score≥1) was also significantly lower in BSSL-KO compared to BSSL-WT male mice (p<0.01 from day 28 onwards; Fig. 1B). At study discontinuation (day 57), only 6 of 17 BSSL-KO male mice (35%) had developed any clinical sign of arthritis, and of these only one had reached an arthritis score>5. In contrast, 14 of 16 BSSL-WT male mice (88%) had developed disease and 13 had reached an arthritis score>5.

In contrast to results obtained with male mice, both the BSSL-KO and the BSSL-WT female mice were highly resistant to CIA and showed consistently low mean arthritis scores (Fig. 1C). Furthermore, the proportion (cumulative incidence) of mice that developed disease (arthritis score≥1) was significantly lower in both BSSL-KO and BSSL-WT female mice compared to BSSL-WT male mice (Fig. 1D). Only four of 12 BSSL-WT (33%) and three of 18 BSSL-KO female mice (17%) showed clinical signs of arthritis at day 57. However, all four diseased BSSL-WT female mice but none of the BSSL-KO female mice had an arthritis score>5 at that point.

**Figure 2:**
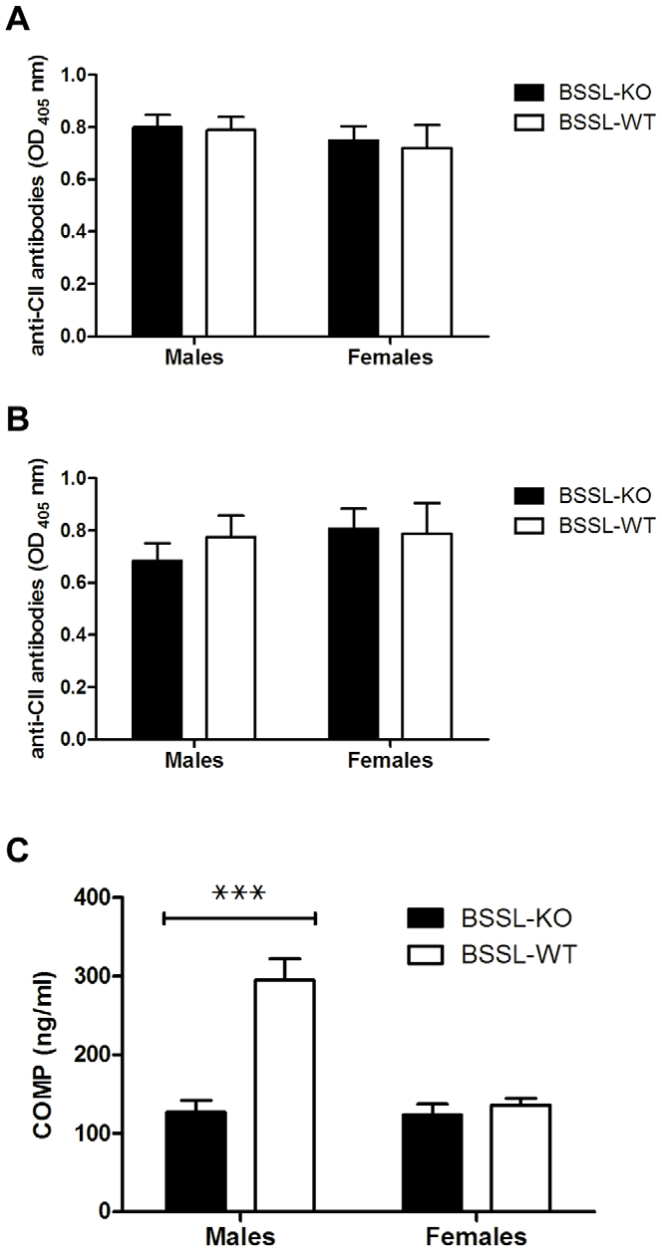
Development of IgG anti-CII antibodies and cartilage degradation in BSSL-KO and BSSL-WT mice. Littermates of BSSL-KO and BSSL-WT mice were immunized with rat CII on day 0 and boosted on day 21 to induce arthritis. The concentration of IgG anti-CII antibody in plasma was determined by ELISA at (A) day 30 and (B) day 57 after initial injection with rat CII. As a marker of cartilage degradation, the concentration of cartilage oligomeric matrix protein (COMP) present in mouse plasma was determined by ELISA (C) on day 57 after initial injection with rat CII. Values are expressed as mean ± SEM. ^???^ = p<0.001.

Concentrations of IgG anti-CII antibody and COMP, as markers of cartilage degradation, were determined in plasma collected on experimental days 30 and 57 (COMP only at day 57). There was no significant difference in antibody response against CII between BSSL-KO and BSSL-WT mice, with no gender differences (Fig. 2A, B). In contrast, significantly lower COMP levels were found in plasma obtained from male BSSL-KO mice compared to male BSSL-WT mice on experimental day 57 (p<0.001; Fig. 2C). Plasma levels of COMP in BSSL-KO and BSSL-WT females were low and comparable to the level found in BSSL-KO male mice.

**Figure 3:**
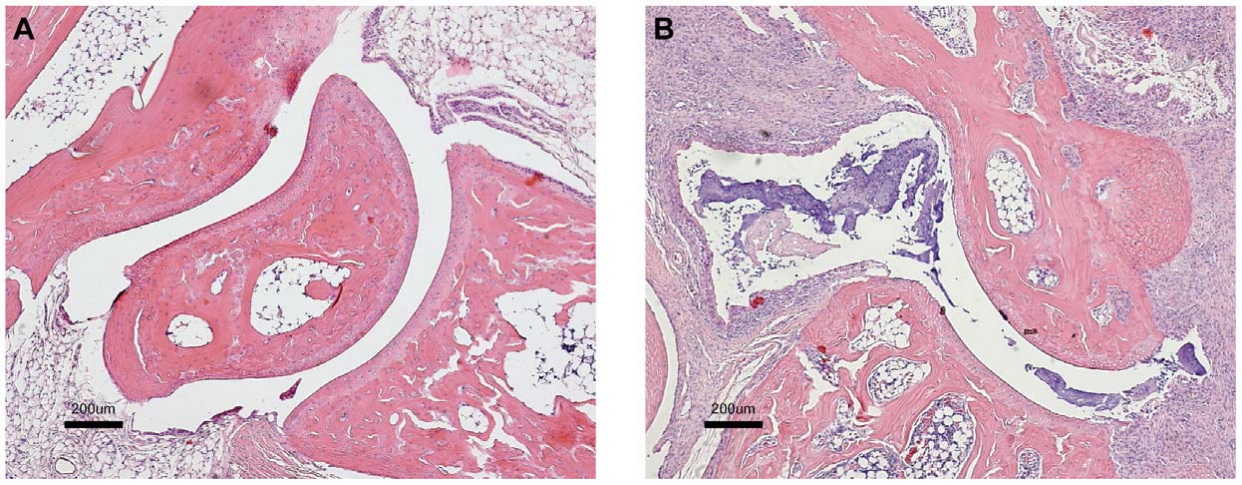
Histology of paw joints from BSSL-KO and BSSL-WT mice. Hematoxylin and eosin staining of hind limb sections from (A) BSSL-KO and (B) BSSL-WT mice taken at day 57 after initial injection with rat CII. The joint of BSSL-KO mice has normal histological appearance without cellular infiltration whereas the joint of BSSL-WT mice shows signs of cartilage destruction and significant inflammatory cell infiltration. Images shown are representative of those obtained from five mice in each group. Original magnification ×4.

To further evaluate arthritis severity, paraffin sections of hind limb ankle joints from five BSSL-WT and five BSSL-KO representative animals were stained with hematoxylin and eosin. A clear association between inflammation score and increased cell infiltration in the joint synovium and cartilage destruction of diseased mice, compared to mice without clinical signs of disease, was observed (Fig. 3).

**Figure 4:**
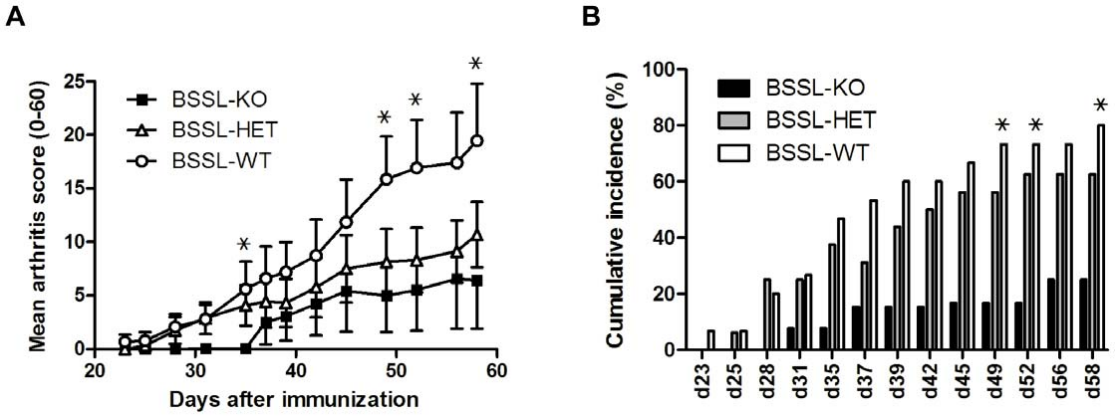
Collagen-induced arthritis in BSSL-KO, BSSL-WT, and BSSL-HET mice. Littermates of male BSSL-KO (n = 13), BSSL-WT (n = 15), and BSSL-HET (n = 16) mice were immunized with chick CII on day 0 and boosted on day 21. Arthritis development was followed by blinded clinical scoring 2–3 times per week for 58 days after initial CII injection. (A) Mean clinical score of arthritis severity and (B) cumulative incidence of arthritis (percentage of affected mice with clinical score≥1) are shown. Error bars indicate ± SEM. ^???^ = p<0.05.

A follow-up study, using essentially the same experimental protocol as above comprised of 13 BSSL-KO, 15 BSSL-WT, and 16 BSSL-HET littermate male mice (age 8–14 weeks), was conducted. The only deviations from the first study protocol were that the mice had been backcrossed to mouse strain C57BL/10Q for seven generations to exclude nonlinked genetic effects and that chick CII, instead of rat CII, was used to induce arthritis. Arthritis scores in this follow-up study were consistent with the previous results obtained for male mice, i.e. BSSL-KO mice developed significantly lower mean arthritis scores compared to their BSSL-WT littermates (p<0.05 on day 35, 49, 52 and 58; Fig. 4A). Furthermore, there was a trend for protection of BSSL-HET mice from developing disease compared to BSSL-WT mice, but they were not as resistant as BSSL-KO mice and the difference between BSSL-HET and BSSL-WT did not reach statistical significance (Fig. 4A). Not only the severity but also the proportion (cumulative incidence) of mice that developed disease (arthritis score≥1) was significantly lower in BSSL-KO compared to BSSL-WT mice (p<0.05 on day 49, 52 and 58; Fig. 4B). It should be noted that the cumulative incidence of disease was also significantly lower in BSSL-KO mice compared to BSSL-HET mice (p<0.05 on day 45 onwards) but after adjusting for multiple testing, these differences did not remain statistically significant. However, at study discontinuation only 3 of 12 BSSL-KO mice (25%) had an arthritis score≥1 compared to 10 of 16 BSSL-HET (62%) and 12 of 15 BSSL-WT littermates (80%).

### BSSL neutralizing antibodies significantly inhibit development of arthritis in mice and rats

**Figure 5:**
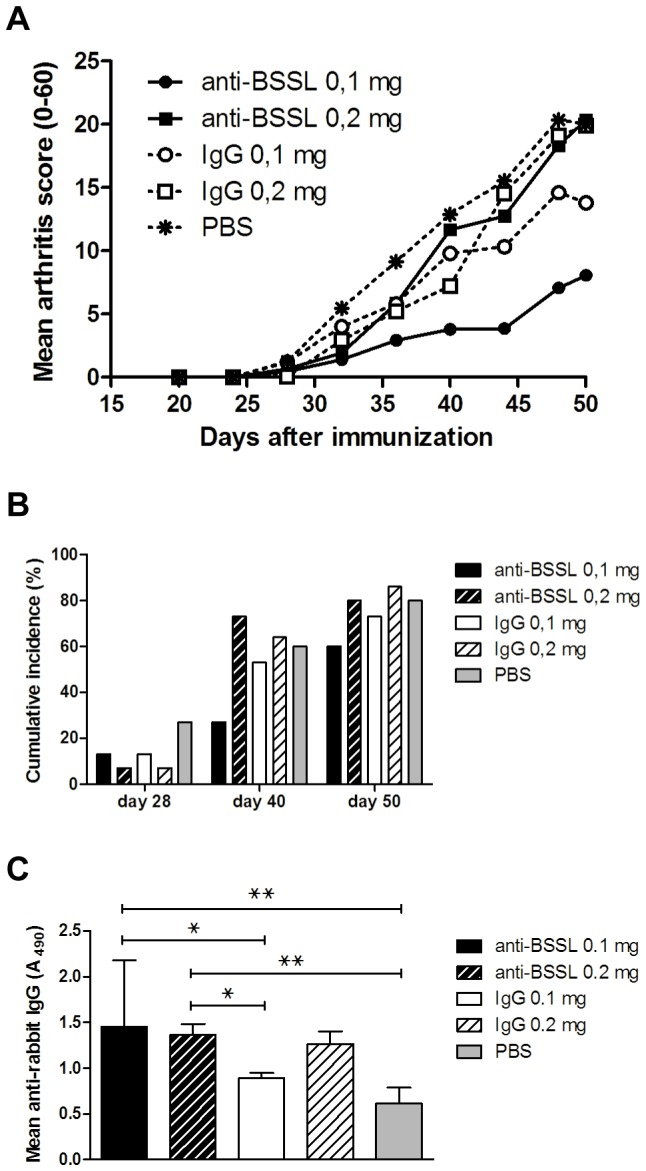
Inhibition of collagen-induced arthritis in DBA/1 mice by treatment with BSSL neutralizing antibodies. Seventy-five DBA/1 female mice were immunized with chick CII in CFA on day 0 and boosted with CII in IFA at day 21. Mice were randomized to one of five groups of 15 animals each, which, beginning on day 20, were treated every fourth day until day 44 with one of the following: 0.1 mg CDV15 antibody, 0.2 mg CDV15 antibody, 0.1 mg normal rabbit IgG, 0.2 mg normal rabbit IgG, or PBS (placebo control). Arthritis development and severity was followed by clinical scoring 2–3 times per week for 50 days after initial CII injection. (A) Mean score of arthritis severity and (B) cumulative incidence of arthritis (percentage of affected mice with clinical score≥1) are shown. (C) At the time of discontinuation (day 50) the concentration of mouse anti-rabbit IgG was determined in plasma collected from all animals. Error bars indicate ± SEM. For clarity, error bars were omitted in (A). ^???^ = p<0.05; ^???^ = p<0.01.

The hypothesis that targeting BSSL with neutralizing antibodies could affect development or severity of CIA was investigated in 75 female DBA/1 mice, randomized to one of five groups (n = 15 per group) for treatment. Two different doses of the anti-BSSL antibody CDV15 were compared to the same doses of normal rabbit IgG and PBS as placebo control. Arthritis was induced in all mice at day 0 and treatment was given as i.p. injections every fourth day, starting the day before boost (day 20). Compared to the placebo control, treatment with 0.1 mg of CDV15 significantly prevented or mitigated development of arthritis at least until the seventh and last dose was given on day 44 (p<0.05 from day 40 onwards if not adjusted for multiple testing; Fig. 5A). Treatment with 0.1 mg normal rabbit IgG also tended to alleviate disease, though less effectively than CDV15. Increasing the dose of CDV15 from 0.1 mg to 0.2 mg per injection did not improve the preventive effect of CDV15 on disease development. In fact, at the time of discontinuation of the study (day 50) the 0.2 mg group showed a mean arthritis score similar to the 0.2 mg normal rabbit IgG and PBS placebo control groups. At day 40 after the initial CII immunization, the relative number of mice that had developed disease (arthritis score≥1) tended to be lower in the group treated with 0.1 mg CDV15 compared to all other groups, although the differences were not statistically significant (p>0.05; Fig. 5B). Nor did the effect obtained by treatment with CDV15 (0.1 mg per injection) on decreased arthritis severity (Fig. 5A) remain statistically significant after adjusting for multiple testing.

To test if the decline in treatment effect seen after the seventh dose of 0.1 mg CDV15 and if the lack of improved efficacy by increasing the CDV15 dose from 0.1 mg to 0.2 mg could be an immunological response to CDV15 resulting in anti-antibody production, the concentration of mouse anti-rabbit IgG was determined in plasma collected from all animals at the time of study discontinuation. Indeed, significantly increased anti-rabbit IgG plasma concentrations were found in the groups treated with 0.1 mg CDV15 and 0.2 mg CDV15 compared to the PBS control group (p<0.01) and to the 0.1 mg normal rabbit IgG group (p<0.05; Fig. 5C).

**Figure 6:**
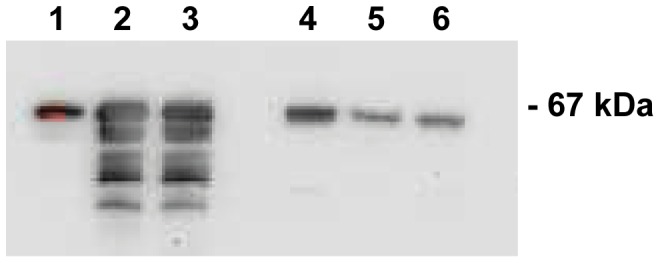
Western blot analysis of rat and mouse pancreas using the CDV15 antibody. Crude extracts derived from three rat pancreata (lane 1–3) and three mouse pancreata (lane 4–6) were separated by SDS-PAGE (12%), transferred to PVDF membranes, and probed with the CDV15 antibody. Partial degradation of the BSSL protein has occurred in lanes 2 and 3. 67 kDa indicates the mass of mouse and rat pancreatic BSSL.

The postulated capacity of BSSL neutralizing antibodies to prevent arthritis was further investigated in arthritic rats. To provide support for the rationale of using the CDV15 antibody in rats we first checked for cross-reactivity of CDV15 with rat BSSL by Western blot. Results showed that although the deduced amino acid sequence of rat BSSL differs at three positions compared to the synthetic peptide used to develop CDV15 antibodies, CDV15 reacted with BSSL present in crude extracts of both mouse and rat pancreas (Fig. 6).

**Figure 7:**
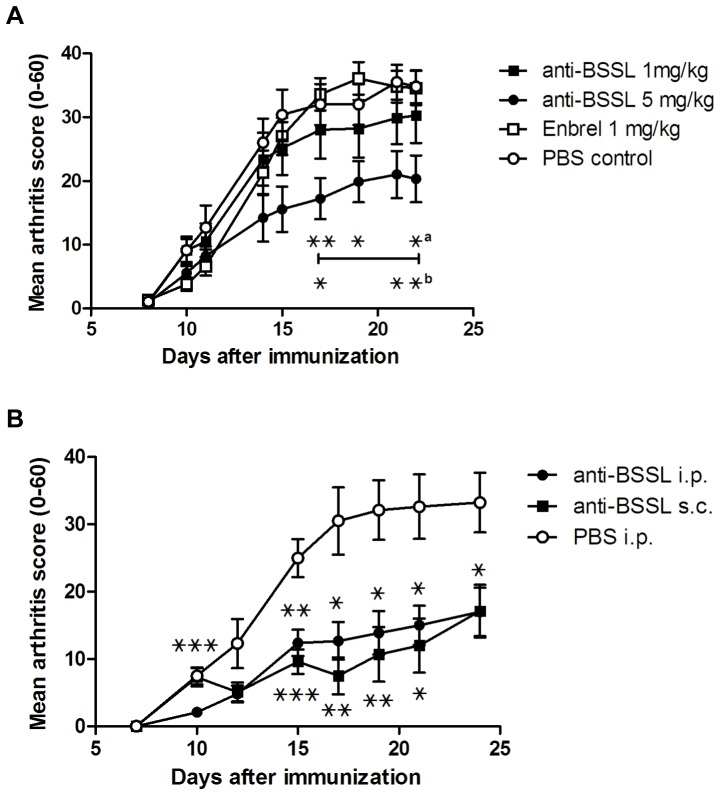
Prevention of PIA in rats by BSSL neutralizing antibodies. DA male rats were immunized with pristane on day 0 to induce arthritis. Rats were randomized to receive different treatments on three separate occasions (day 5, 10, and 15) as follows: (A) Four groups of rats (n = 10 each) were treated with i.p. injection of either CDV15 antibody 1 mg/kg, CDV15 antibody 5 mg/kg, etanercept 1 mg/kg, or PBS (placebo control). (B) Three groups of rats (n = 10 each) were treated with one of the following: i.p. injection of CDV15 antibody 5 mg/kg, s.c. injection of CDV15 antibody 5 mg/kg, or PBS (i.p. injection). Arthritis development was followed by clinical scoring 2–3 times per week for 23 or 24 days after initial pristine injection. Mean scores of arthritis severity are shown. Error bars indicate ± SEM. ^???^ = p<0.05; ^???^ = p<0.01; ^???^ = p<0.001.

Forty male DA rats (age 8–10 weeks), randomized to one of four groups (n = 10 per group) for treatment, were given a single injection of pristane, known to induce arthritis within two weeks [24]. Treatment was given as i.p. injections (1 ml) on days 5, 10, and 15 after pristane immunization. Two different doses of CDV15 antibody were tested (1 mg/kg and 5 mg/kg) and the effect was compared to treatment with etanercept (1 mg/kg) or PBS as placebo control. Clinical scoring showed that although the incidence of disease development (arthritis score≥1) was 100% in all groups (data not shown), treatment with CDV15 anti-BSSL antibodies at the higher dose (5 mg/kg per injection) significantly reduced disease severity as compared to both etanercept and PBS (p<0.05 from day 17 onwards; Fig. 7A). Additionally, the group receiving the lower dose of CDV15 (1 mg/kg per injection) showed a clear tendency towards amelioration, supporting a dose-dependent preventive effect. The protective effect of anti-BSSL treatment on the clinical severity of arthritis was confirmed by histological examination. Treated animals showed a marked decrease in the number of inflammatory cells present in the joint synovium and less cartilage destruction (data not shown).

To verify the results obtained by injecting anti-BSSL CDV15 antibody during PIA in rats and at the same time compare different routes for treatment, a follow-up study using essentially the same experimental protocol as above was conducted. Thirty male DA rats (age 8–10 weeks) were randomized into three groups (n = 10 per group) for treatment. CDV15 antibody (5 mg/kg) was injected either i.p. or s.c. on days 5, 10, and 15 after pristane immunization and the effect was compared to i.p. PBS as placebo control. Clinical scoring confirmed previous results and showed that regardless of injection route, treatment with CDV15 anti-BSSL antibody (5 mg/kg per injection) significantly reduced disease severity as compared to PBS (p<0.05 from day 15 onwards; Fig. 7B).

## Discussion

Novel observations presented here strongly support our hypothesis that vascular BSSL, originating in neutrophils and platelets, affects inflammatory conditions such as chronic arthritis. We applied the CIA model and in two consecutive studies demonstrated that BSSL-KO mice, in contrast to BSSL-WT littermates, were significantly protected from developing inflammatory arthritis defined by swelling and redness of the paw joints. BSSL-KO mice developed arthritis at a lower incidence and reduced severity compared to BSSL-WT littermates. We also showed that BSSL-HET mice were less prone to develop disease as compared to BSSL-WT mice, but they were not as resistant as BSSL-KO mice, suggesting a gene-dose effect. In one of the studies we noticed a gender-based difference when only four of 12 female BSSL-WT mice (33%) compared to 14 of 16 male BSSL-WT mice (88%) developed disease. This was not surprising, since it is well known that males are more often affected than females in the CIA mouse model [36]. Results from clinical scoring were confirmed by histological evaluation. Sections of hind limbs obtained from diseased mice showed massive infiltration of inflammatory cells in the joint synovium and cartilage destruction. These features were not detected in samples collected from mice without clinical signs of disease.

It should be noted that BSSL-KO mice show normal growth and development and, besides impaired intestinal absorption of esterified cholesterol due to the enzyme's cholesteryl ester hydrolyzing activity [37], adult mice do not suffer from increased morbidity [25], [38], [39]. So far, the only reported notable negative phenotype associated with BSSL deficiency is restricted to newborn BSSL-KO pups, which exhibit significantly reduced capacity to digest dietary milk fat compared to BSSL-WT pups. As a result, lipid droplets accumulate in the distal small intestine and injure the villus epithelium [40]. However, this is a transient phenotype that disappears during the suckling-weaning transition as expression of pancreatic lipase and phospholipase A2, the two major enzymes responsible for intestinal digestion of dietary fat in adults, increase and thus compensate for the lack of BSSL [9], [41].

Currently, we can only speculate on the mechanism through which BSSL influences the inflammatory process. As there was no difference between BSSL-KO and BSSL-WT mice in the CIA model with respect to anti-CII IgG levels it is unlikely that BSSL affects B cell activity. Since BSSL, as already mentioned, is proposed to interact with CXCR4 on platelets [23], one appealing possibility is that BSSL is involved in recruitment of inflammatory cells via CXCR4 and its ligand chemokine stromal cell-derived factor 1 (SDF-1, also termed CXCL12). Very recently, BSSL was identified as a marker for HIV-1 progression to AIDS and emergence of CXCR4-using viruses in HIV-1 infected patients. Furthermore, an association between *BSSL* genotypes and CD4 cell count in blood of uninfected individuals was reported [42]. Several studies suggest that the SDF-1/CXCR4 axis plays a central role in the pathogenesis of RA by triggering migration and recruitment of leukocytes, activated T cells, and plasmacytoid dendritic cells into the inflamed joints [43]–[47]. *Cxcr4* and *Cxcl12* null-knockout mice are embryonic lethal [48], [49] but studies using T cell-specific CXCR4-deficient mice showed that CXCR4 expression in T cells is important for the development of CIA by recruiting activated T cells toward inflammatory sites [46]. In patients with RA the frequency of CXCR4 expressing synovial tissue CD4^+^ memory T cells is elevated compared with normal controls [50] and the concentration of SDF-1 in synovial fluid is significantly increased [51].

Previous studies found that treatment with AMD3100, a specific CXCR4 antagonist, significantly reduced the severity of CIA in mice [43], [44]. AMD3100 was further shown to inhibit the chemotactic effect of SDF-1 on splenocytes *in vitro* as well as migration of leukocytes into SDF-1 containing subcutaneous air pouches *in vivo* [44]. In the present study we found evidence that BSSL-neutralizing antibodies significantly reduced the incidence and severity of CIA and PIA in mice and rats, respectively. The unexpected reverse dose-response effect in the mouse CIA experiment could be explained by an immunological response to CDV15 resulting in anti-antibody production towards rabbit IgG. This was confirmed by higher anti-rabbit IgG levels in the IgG treated groups compared to the PBS control group. It seems reasonably that a more pronounced difference between groups (0.1 mg vs. 0.2 mg) had been observed had the evaluation been made before the effect started to decrease at day 44 in the CDV15 0.1 mg group.

In contrast to AMD3100 it is unlikely that BSSL antagonizes SDF-1/CXCR4 signaling. We rather hypothesize that BSSL interferes with the interaction of SDF-1 with CXCR4 and thus triggers the signaling pathway leading to migration and recruitment of inflammatory cells to the site of acute inflammation. This could initially be a positive role for BSSL, but when the inflammation is no longer controlled and becomes “chronic”, BSSL may sustain the inflammatory response and hence become a negative factor and a target for treatment of inflammatory disease.

Taken together, the present data support a key role for BSSL in the inflammatory process, at least in rodents. In the future, more studies are warranted to explore the exact mechanism by which BSSL is involved in the inflammatory process, and whether a BSSL-neutralizing agent could serve as a therapeutic model to reduce the inflammatory response in humans as well.
